# Towards tailoring blood pressure control in HFpEF: Lessons from OPTIMIZE-HF

**DOI:** 10.21542/gcsp.2019.3

**Published:** 2019-03-31

**Authors:** Mohamed Hassan

**Affiliations:** Aswan Heart Centre, Aswan, Egypt

## Introduction

Heart failure (HF) with preserved ejection fraction (HFpEF) represents approximately 50% of the world’s HF population, and this proportion is increasing over time^1^. The diagnosis of HFpEF is more challenging than HF with reduced ejection fraction (HFrEF). Patients with HFpEF are significantly older, more likely to be female, and more likely to have hypertension, obesity, anemia, atrial fibrillation, renal disease, and pulmonary disease compared to those with HFrEF^2,3^. In observational studies, rates of hospitalization and death among patients with HFpEF approach those with HFrEF^4,5^, however in clinical trial populations, outcomes are better in patients who have HFpEF^6^. Death from non-cardiovascular causes is more common in patients who have HFpEF than in those with HFrEF, and a smaller percentage of patients with HFpEF die from CVD-related causes^7^.

Hypertension has been identified as a major risk factor for incident HF, and treatment of hypertension, particularly to a systolic blood pressure (SBP) target level of less than 120 mmHg, has been demonstrated to substantially lower the risk of incident HF ^8,9^. However, the optimal SBP target level is less clear for patients with HFpEF. The recent American College of Cardiology/American Heart Association (ACC/AHA) guidelines for HF recommends new SBP target goal - less than 130 mmHg - for patients with HFpEF and persistent hypertension after management of volume overload^10^. However, once patients develop HF, a lower SBP level may have a paradoxical effect. Several studies have demonstrated increased cardiovascular (CV) and HF mortalities, as well as all-cause, CV, and HF hospitalizations when baseline SBP ≤ 120 mmHg independent of other baseline characteristics^11,12^. However, little data exits regarding this association in patients with HFpEF.

## The study

This is a propensity score matched observational study of the Medicare-linked Organized Program to Initiate Lifesaving Treatment in Hospitalized Patients with Heart Failure (OPTIMIZE-HF) registry that included 25,354 patients who were discharged alive. The study was subsequently published in The Journal of the American Medical Association (JAMA)-Cardiology^13^.

The analysis was restricted only to 3,915 patients who have HFpEF (Ejection fraction (EF) at least 50%) with stable in-patient SBP levels, defined as an admission to discharge variation ≤ 20 mmHg (median = −4). Among these patients, 901 (83.7%) with discharge SBP <120 mmHg were matched by propensity scores with 901 patients with SBP levels ≥ 120 mmHg who were balanced on 58 baseline characteristics. A SBP cutoff of 120 was chosen because SBP level <120 mmHg has been shown to be associated with poor outcomes in HF^11,14^.

The primary outcomes included all-cause mortality and HF re-admission at 30 days, 1 year, and during overall median follow-up of 2.1 (overall 6) years. Secondary outcomes included all-cause readmission and the 2 combined end points of all-cause readmission or all-cause mortality and HF readmission or all-cause mortality.

**Figure 1.**
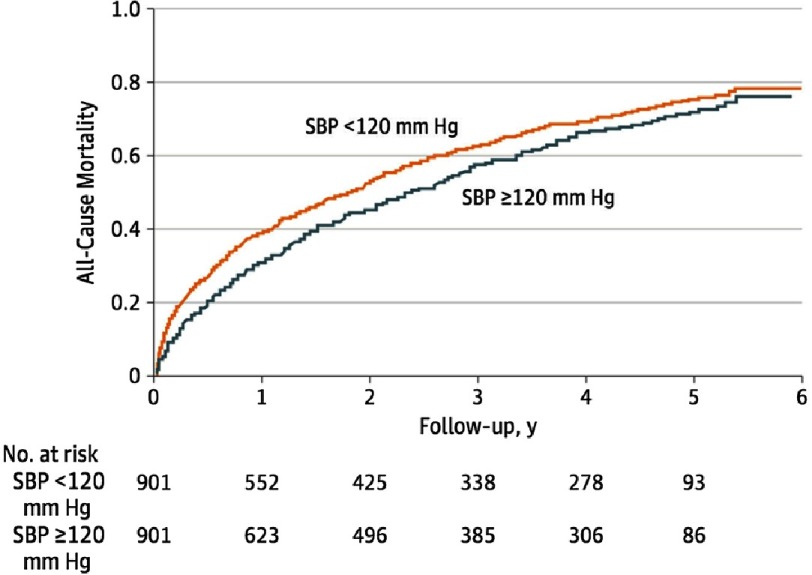
Kaplan-Meier plot for all-cause mortality in 901 pairs of propensity score–matched patients with HFpEF, by SBP <120 vs ≥120 mm Hg.

The 1,802 matched patients had a mean age of 79 ± 10 years and a mean EF of 59% ± 7%. SBP level was less than 90 mmHg in only 13 matched patients (2 had <80 mmHg). The mean diastolic BP was 60 mmHg for patients with discharge SBP <120 and 69 mmHg for those with discharge BP ≥ 120 mmHg. The 30-day all-cause mortality occurred in 91 (10%) of matched patients with discharge SBP <120 mmHg compared to 45 (5%) of those with discharge SBP ≥ 120 mmHg (hazard ratio [HR] = 2.07; 95% confidence interval CI [1.45–2.95]; *P* <.001). SBP <120 mmHg was also associated with a higher risk of mortality at 1 year (39% vs 31%; HR = 1.36; 95% CI [1.16–1.59]; *P* <.001) and during a median follow-up of 2.1 years (HR = 1.17; 95% CI [1.05–1.30]; *P* = .005) (Figure 1).

**Figure 2.**
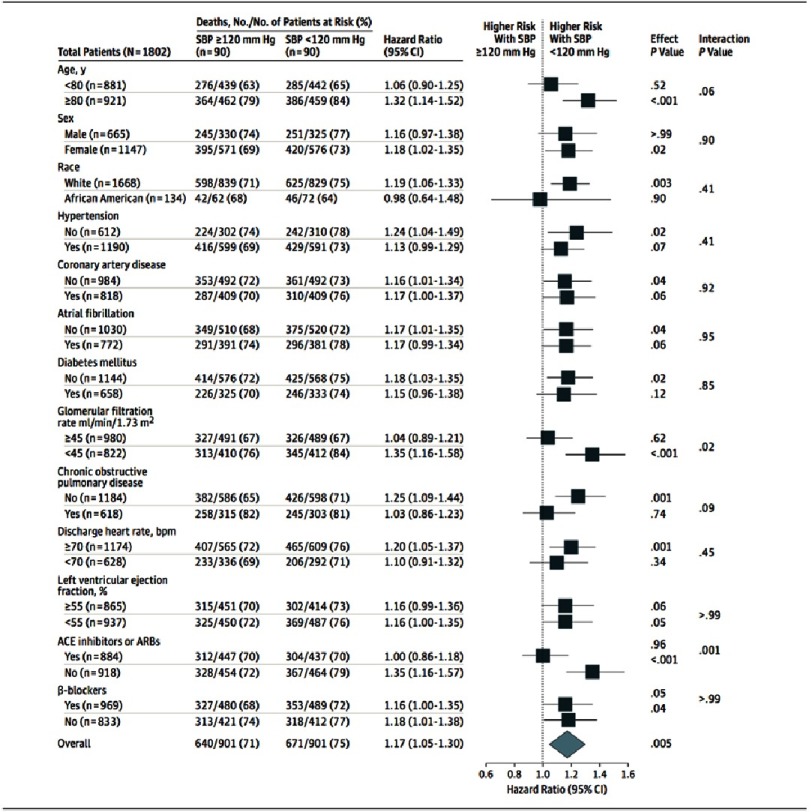
Forest plots for subgroup analyses of mortality by SBP level.

Furthermore, SBP <120 mmHg was associated with a higher risk of HF readmission at 30 days (HR= 1.47; 95% CI [1.08–2.01]; *P* = 0.02) but not at 1 or 6 years. In addition, the combined end point of HF readmission or all-cause mortality at 30 days, 1 year, and overall follow up period were significantly higher in patients with SBP <120 mmHg compared to those with SBP ≥ 120 [HR = 1.71 (95% CI [1.34–2.18]; *P* <0.001), 1.21 (95% CI [1.07–1.38]; *P* = 0.004), and 1.12 (95% CI [1.01–1.24]; *P* = 0.03), respectively]. These associations were homogenous across various clinically relevant subgroups of patients, except those with a glomerular filtration rate ≥ 45 mL/min/1.73 m^2^ and those who received a discharge prescription for ACE inhibitors (Figure 2). Similar results were obtained when a SBP cutoff of 130 mmHg was used.

## Discussion

The current study demonstrated a consistent association between lower SBP levels and higher mortality a well as higher risk of re-hospitalization for HF in patients with HFpEF. SBP below 120 mmHg at discharge was associated with double risk of death within 30 days of discharge and a 36% increased one-year mortality risk. Discharge SBP below 130 mmHg was also associated with poor outcomes, though not to the extent of levels below 120 mmHg. This could be a reflection more advanced disease state and lower cardiac output in patients with lower discharge SBP levels.

The adverse events in the African American subgroup of patients –which represent only 7–8% of the study patients –were similar in both BP lowering strategies. Hypertension is a major problem in African American patients and usually follows a more aggressive course, and is associated with a higher risk of CV complications compared to age- and BP-matched white hypertensive patients. Whether this particular subgroup warrants a more aggressive BP lowering strategy –in view of their higher risk –needs to be further tested in larger prospective studies.

Although the current guidelines recommend achieving SBP <130 mmHg in patients with HFpEF and persistent hypertension, the current study revealed poor outcomes when this target was achieved. In fact, these recommendations are not extrapolated from HF populations. Moreover, ACE inhibitors and angiotensin II receptor blockers did not improve the outcome in patients with HFpEF, in spite of their blood pressure lowering effect^15,16^.

All these findings were based on discharge SBP only. Unfortunately, no data is available regarding the post discharge SBP level, and SBP crossover during follow-up period which might change the results. It should be noted also that the current findings are applicable only in hospitalized patients with HFpEF, and cannot be generalized to ambulatory patients because the determinants of blood pressure in these 2 settings are different.

## What have we learned?

Aggressive lowering of blood pressure in older patients with HFpEF needs to be avoided. Among hospitalized older patients with HFpEF, a discharge SBP of less than 120 mmHg – or even less than 130 mmHg –is associated with poor short- and long-term cardiovascular outcomes. Future prospective studies are needed to evaluate optimal SBP treatment goals in patients with HFpEF.
